# Prevalence and Associated Factors of Thyroid Dysfunction Among Clinical Populations in Bangladesh: A Systematic Review and Meta‐Analysis

**DOI:** 10.1002/puh2.70446

**Published:** 2026-11-07

**Authors:** Most. Rubaia Akter Rifat, Md. Raiyan Hasan Nabid, Md. Tanjim, Nourin Sultana, Md. Moin Uddin Riyad, Md. Kaderi Kibria

**Affiliations:** ^1^ Department of Statistics Hajee Mohammad Danesh Science and Technology University Dinajpur Bangladesh; ^2^ Department of Statistics and Data Science University of Rajshahi Rajshahi Bangladesh; ^3^ Department of Computer Science and Engineering University of Asia Pacific Dhaka Bangladesh

**Keywords:** associated factors, Bangladesh, hyperthyroidism, hypothyroidism, meta‐analysis, metabolic syndrome, prevalence, thyroid dysfunction

## Abstract

Thyroid dysfunction (TD) is a significant endocrine burden with profound implications for systemic health; however, consolidated epidemiological data within the clinical landscape of Bangladesh remain sparse. This systematic review and meta‐analysis aimed to estimate the pooled prevalence and identify clinical factors associated with TD among Bangladeshi clinical populations using data published between 2010 and 2025. Following Preferred Reporting Items for Systematic Reviews and Meta‐Analyses (PRISMA) guidelines and PROSPERO registration (CRD42025116168), six electronic databases were searched without language restrictions. Data from clinical populations (aged ≥12 years) were synthesized using a random‐effects model. Subgroup analysis and univariable random‐effects meta‐regression were employed to explore drivers of heterogeneity, whereas publication bias was assessed via Peters’ regression test. The pooled prevalence of TD was 23.72% (95% confidence interval [CI]: 17.90–30.72) across clinically heterogeneous populations, with hypothyroidism identified as the predominant abnormality (20.55%) over hyperthyroidism (4.93%). Meta‐regression identified clinical profile as the factor most strongly associated with heterogeneity, revealing that patients with metabolic‐inflammatory disorders (e.g., diabetes, metabolic syndrome) exhibited a markedly higher prevalence (34.77%) than endocrine‐reproductive groups (19.14%). Furthermore, comprehensive biochemical assessments (thyroid‐stimulating hormone [TSH] with free hormones) yielded higher prevalence estimates (28.31%) than limited TSH‐only testing (18.31%). Peters’ regression test showed no significant asymmetry (*p* = 0.384), though this should be interpreted cautiously given the small number of studies (*k* = 8). These results underscore a critical “dual burden” of endocrine and systemic disease in Bangladesh. The high prevalence within metabolic high‐risk groups necessitates the integration of routine, comprehensive thyroid screening into standard clinical management protocols to optimize endocrine care and mitigate long‐term systemic complications.

## Graphical abstract

This systematic review and meta‐analysis of Bangladeshi clinical populations found that nearly one‐quarter (23.72%) had thyroid dysfunction, predominantly hypothyroidism. Patients with metabolic‐inflammatory disorders showed markedly higher prevalence (34.77%) than endocrine‐reproductive groups (19.14%), highlighting a “dual burden” of endocrine and systemic disease requiring integrated thyroid screening in high‐risk clinical care.

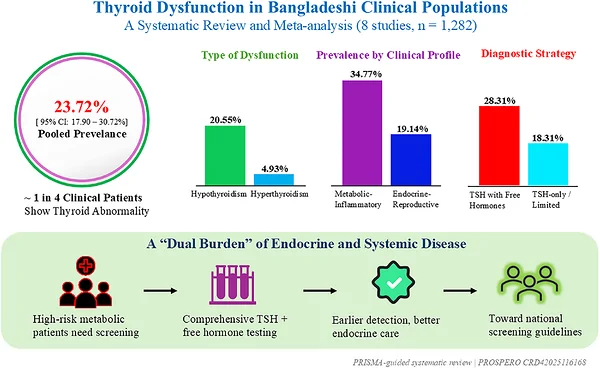

## 1 Introduction

Thyroid dysfunction (TD) comprises a spectrum of endocrine disorders resulting from abnormalities in thyroid hormone synthesis, secretion, or peripheral action. Ranked as the second most prevalent endocrine condition globally after diabetes mellitus, TD poses a substantial challenge to global health systems [1]. The thyroid gland, through the secretion of thyroxine (T4) and triiodothyronine (T3), serves as a master regulator of basal metabolic rate, cardiovascular hemodynamics, thermogenesis, and neurocognitive development [2, 3, 4, 5]. Consequently, the disruption of thyroid homeostasis precipitates systemic manifestations ranging from neuropsychiatric disturbances and menstrual irregularities to life‐threatening cardiovascular and metabolic complications [6, 7]. The clinical landscape of TD is traditionally bifurcated into hypothyroidism and hyperthyroidism, occurring in both overt and subclinical forms [8]. Although overt dysfunction presents with distinct symptomatic profiles such as bradycardia and cognitive slowing in hypothyroidism or tachyarrhythmias and tremors in hyperthyroidism, subclinical TD is increasingly recognized as a “silent” clinical priority. Characterized by abnormal thyroid‐stimulating hormone (TSH) levels despite normal circulating free hormones (FT4, FT3), subclinical states are strongly associated with long‐term cardiovascular risks, metabolic syndrome, and adverse obstetric outcomes [9, 10].

The epidemiology of TD is highly dynamic, influenced by an interplay of autoimmune triggers, iodine nutrition, genetic susceptibility, and environmental exposures [11]. Globally, prevalence estimates range from 4% to 10% in adult populations, with significant variations dictated by age, sex, and geographic location [12, 13, 14, 15]. In South Asia, the burden of thyroid disorders is particularly complex. Bangladesh has undergone rapid demographic transitions and urbanization, leading to a “double burden” of disease where nutritional deficiencies coexist with rising metabolic non‐communicable diseases (NCDs) [12]. Although the universal salt iodization (USI) program has successfully mitigated iodine deficiency disorders, a paradoxical shift has been observed where optimal or excessive iodine intake, coupled with lifestyle changes, may be driving a higher incidence of autoimmune‐mediated dysfunction [16]. Despite the escalating clinical recognition of TD in Bangladesh, a significant conceptual and evidence gap persists. Existing literature is characterized by fragmented, region‐specific studies with small sample sizes and heterogeneous diagnostic thresholds [12, 17, 18, 19, 20]. Furthermore, there is a lack of high‐level synthesis regarding the determinants of TD in clinical high‐risk groups. However, primary studies hint at a high prevalence among patients with metabolic and inflammatory disorders, reflecting a biological “crosstalk” between insulin resistance and thyroid signaling. This relationship has not been quantitatively consolidated in the Bangladeshi context. This lack of pooled evidence obscures the true disease burden, hinders the identification of high‐risk subgroups, and prevents the formulation of standardized national screening guidelines. This study provides the first comprehensive systematic review and meta‐analysis to quantify the pooled prevalence of TD in Bangladesh. Beyond mere estimation, this research employs meta‐regression to investigate the clinical determinants of prevalence variability, focusing on the intersection of metabolic health and thyroid function. The findings of this study carry profound implications for public health policy and the achievement of the United Nations Sustainable Development Goals (SDGs), specifically Goal 3 (Good Health and Well‐being) [21, 22]. By providing robust epidemiological data, this research supports the integration of thyroid screening into primary healthcare and NCD management protocols [12]. Reducing the burden of undiagnosed TD is essential for decreasing premature mortality from NCDs, including cardiovascular diseases and diabetes complications, thereby strengthening the national endocrine care framework and supporting evidence‐based resource allocation in Bangladesh.

## 2 Materials and Methods

This systematic review and meta‐analysis were conducted in strict accordance with the Preferred Reporting Items for Systematic Reviews and Meta‐Analyses (PRISMA) guidelines to ensure transparency, methodological rigor, and reproducibility [23]. The study protocol was prospectively registered in the PROSPERO database (Registration number: CRD42025116168).

### 2.1 Search Strategy and Study Identification

A systematic and comprehensive literature search was executed across six major electronic databases: PubMed, Google Scholar, CORE, Europe PMC, Scopus, and the Bangladesh Journal of Medicine. To ensure maximum coverage, the search was supplemented by a manual review of gray literature, government reports, institutional repositories, and conference proceedings. The search string utilized a combination of Boolean operators and MeSH terms: ((“thyroid dysfunction” OR “hypothyroidism” OR “hyperthyroidism” OR “thyroid disorder” OR “thyroid disease” OR “thyroid prevalence”) AND “Bangladesh”). The search encompassed studies published between 2010 and 2025 without language restrictions, ensuring a comprehensive capture of recent epidemiological trends influenced by rapid urbanization, nutritional transitions, and evolving healthcare accessibility. Following the removal of duplicate records, two independent reviewers screened titles and abstracts against the eligibility criteria. Full‐text articles were then independently assessed for inclusion. Any discrepancies were resolved through consensus or via consultation with a third senior reviewer.

### 2.2 Inclusion and Exclusion Criteria

Study eligibility was determined using the PCOS (Population, Comparison, Outcome, and Study design) framework to ensure relevance and methodological quality [24]. The population of interest included individuals aged ≥12 years residing in Bangladesh. The primary outcome was the prevalence of TD, including hypothyroidism, hyperthyroidism, and other clinically diagnosed thyroid abnormalities. Studies employing standard thyroid function testing (TSH alone, TSH with total T4, or TSH with free hormones [FT4/FT3]) were eligible for inclusion, provided explicit prevalence data for TD were reported. To ensure study uniformity, peer‐reviewed cross‐sectional studies published between 2011 and 2025 were included. Studies were excluded if they were conducted outside Bangladesh, lacked explicit prevalence data or original primary data, did not differentiate TD from other metabolic or hormonal conditions, or were reviews, case reports, or editorials.

### 2.3 Quality Assessment and Risk of Bias

The methodological quality of the eligible studies was evaluated using the NOS, adapted for cross‐sectional studies [25]. Each study was appraised across three critical domains: selection of study cohorts, comparability of groups, and ascertainment of the outcome. Two reviewers independently assigned scores, with a third reviewer resolving any disagreements. Studies were categorized based on their scores as high, moderate, or low quality, ensuring that only methodologically sound evidence contributed to the quantitative synthesis.

### 2.4 Data Extraction

Data were systematically extracted into a standardized and piloted Excel template. The extracted parameters included: primary author and publication year; data collection period; study setting and geographical region (categorized as “Dhaka” or “Outside Dhaka”); sample size; participant demographics (age and sex); diagnostic criteria; and the specific prevalence of hypothyroidism and hyperthyroidism. All extraction procedures were performed in duplicate to minimize errors and ensure data integrity.

### 2.5 Outcome Measures

The primary outcome of this study was the pooled prevalence of TD among clinical populations in Bangladesh, defined by abnormal biochemical levels of TSH, FT4, and/or FT3. Secondary outcomes involved stratified analyses to investigate the factors associated with prevalence variability. These included the clinical profile, comparing metabolic‐inflammatory disorder populations against endocrine‐reproductive populations; the diagnostic assessment strategy, distinguishing between comprehensive hormone testing (TSH with free hormones) and basic (TSH‐only or limited) assessments; and the specific type of TD, comparing the pooled prevalence of hypothyroidism versus hyperthyroidism. Additionally, the influence of study characteristics was evaluated by stratifying findings according to a median sample size threshold (*n* = 155) to assess the impact of study scale on the pooled estimates. For all measures, prevalence was calculated as the ratio of individuals diagnosed with TD to the total number of participants within the respective study or subgroup. As the included studies drew from diverse clinical populations, the overall pooled prevalence should be interpreted as a composite indicator of TD burden across clinical settings in Bangladesh, rather than the prevalence within any single patient population.

### 2.6 Statistical Analysis

Statistical synthesis was performed using R software (v4.5.1) with the *meta* and *metafor* packages [26]. The pooled prevalence and corresponding 95% confidence intervals (CIs) were calculated using the *metaprop* function. Given the anticipated clinical and methodological diversity across studies, a random‐effects model (DerSimonian and Laird) was employed to account for between‐study variability [27, 28]. Statistical heterogeneity was quantified using Cochran's Q test and the *I* ^2^ statistic, where *I* ^2^ > 75% and *p* < 0.05 indicated substantial heterogeneity [29, 30]. To investigate potential sources of heterogeneity, univariable random‐effects meta‐regression and subgroup analyses were conducted using the *metareg* function. Publication bias was assessed visually via funnel plots and statistically through Peters’ regression test, which is preferred for proportion‐based meta‐analyses [31, 32]. Finally, the stability of the pooled estimates was validated through a leave‐one‐out sensitivity analysis, sequentially excluding each study to determine its individual influence on the overall results [33]. Given the limited number of included studies (*k* = 8), meta‐regression analyses were considered exploratory; *R* ^2^ values were interpreted as indicative of relative covariate contribution rather than precise estimates of explained heterogeneity, consistent with recommendations that meta‐regression is underpowered when *k* < 10 [34, 35].

## 3 Results

### 3.1 Literature Search and Study Selection

A comprehensive search across six electronic databases initially identified 1282 potentially relevant records. Following the removal of 115 duplicates, 1167 unique studies underwent title and abstract screening. During this primary phase, 925 studies were excluded for being irrelevant or failing to meet predefined inclusion criteria. Of the remaining 242 studies, 230 full‐text articles were successfully retrieved, whereas 12 could not be accessed.

Following a rigorous full‐text evaluation of the 230 retrieved articles, 222 studies were excluded for the following reasons: 152 were outside the study's scope, 62 were review articles, and 8 utilized inappropriate study designs. Ultimately, 8 studies met all eligibility requirements and were included in the final quantitative synthesis [36, 37, 38, 39, 40, 41, 42, 43]. The complete study selection process is illustrated in the PRISMA flow diagram (Figure 1).

**FIGURE 1:**
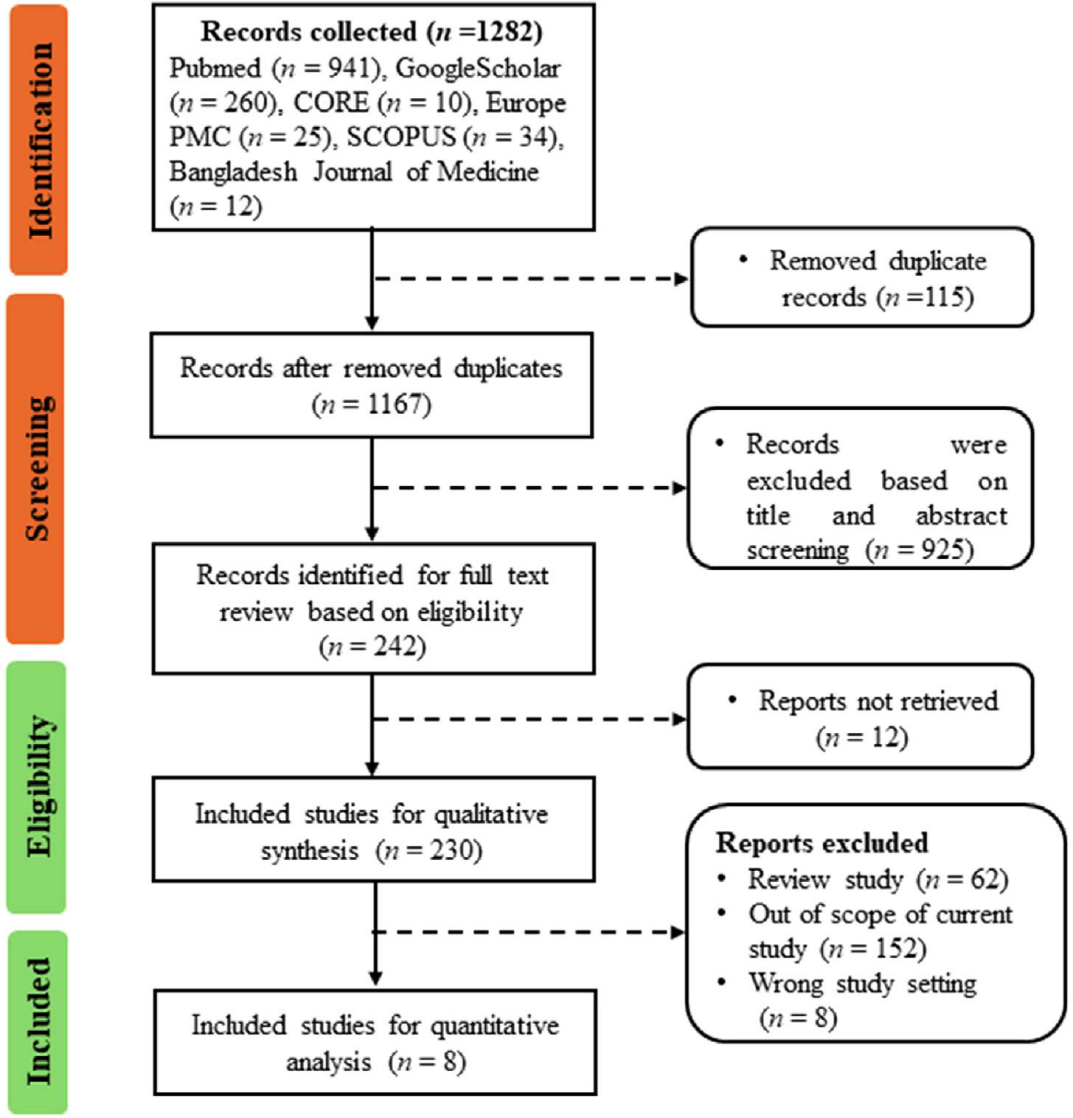
PRISMA flow diagram illustrating the systematic literature search, screening, and selection process for thyroid dysfunction studies in Bangladesh.

### 3.2 Characteristics of Included Studies

The baseline characteristics of the eight included studies are summarized in Table 1. All eligible studies were cross‐sectional in design and published between 2009 and 2025. Geographically, the investigations were primarily concentrated in Dhaka and Sylhet, encompassing both urban and rural communities. The diagnosis of TD across these studies was primarily based on biochemical assays measuring TSH, FT4, and FT3, with a subset of studies also evaluating total T4. Sample sizes ranged from 100 to 323 participants, yielding a pooled total of 1411 individuals. The study populations comprised adults of both sexes, though several studies focused specifically on women of reproductive age. Notable variation was observed in the reported prevalence of TD, likely attributable to differences in study populations, varying clinical comorbidities, and distinct diagnostic thresholds. Clinically, hypothyroidism appeared more frequently than hyperthyroidism across the majority of the cohorts. Therefore, the included data represent a diverse range of clinical settings in Bangladesh, including patients with diabetes mellitus, metabolic syndrome, infertility, rheumatoid arthritis, and those undergoing general endocrine evaluations.

**TABLE 1:** Baseline characteristics of the included studies (*n* = 8) assessing the prevalence of thyroid dysfunction in clinical populations of Bangladesh.

References	Region	Year of data collection	Study type	Total	Age years	Sex (F/M)	Mean age years	Hypothyroid	Hyperthyroid	Thyroid dysfunction	Male	Female	Thyroid diagnosis criteria
[36]	Sylhet	2009–2010	CS	204	≥25	F/M	NA	38	NA	38	10	28	TSH and T4 levels
[39]	Dhaka	2023	CS	140	18–75	F/M	46.7 ± 12.4	38	16	54	NA	NA	TSH, FT4, FT3 levels
[40]	Dhaka	2010–2011	CS	150	23–34	F	NA	26	3	29	NA	29	TSH, FT4, FT3 levels
[41]	Dhaka	2016	CS	160	18–45	F	NA	27	7	34	NA	34	TSH, FT4, FT3 levels
[42]	Dhaka	2018–2019	CS	100	18–70	F/M	47.2 ± 11.3	19	7	26	4	22	TSH, FT4, FT3 levels
[43]	Urban, Dhaka	2014	CS	232	≥21	F/M	45	45	NA	45	NA	NA	TSH levels
[37]	Dhaka	2018–2023	CS	323	19–84	F/M	NA	120	NA	120	NA	NA	TSH, FT4 levels
[38]	(Urban, Rural)	2019	CS	102	≥20	(F/M)	45.1 ± 7.4	12	2	14	NA	NA	NA

*Note:* Diagnostic testing varied across studies (TSH‐only, TSH + T4, TSH + FT4/FT3); this variation is addressed in the diagnostic‐strategy subgroup analysis (Section 3.5) and discussed as a limitation (Section 4.1).

Abbreviations: CS, cross‐sectional study; NA, not reported in source study.

### 3.3 Risk of Bias Assessment

The methodological quality of the eight studies included in this meta‐analysis was evaluated using the NOS (Table S1). Overall quality scores ranged from 6 to 8 out of a possible 9, indicating that all included investigations demonstrated acceptable methodological rigor. One study achieved a score of 8, representing high quality, whereas the remaining seven studies scored between 6 and 7, reflecting moderate quality. Most studies utilized appropriate outcome assessments and statistical analyses; however, minor limitations were identified regarding representativeness and response rates due to limited reporting of sampling procedures. No studies were rated as low quality, confirming their suitability for inclusion in the quantitative synthesis.

### 3.4 Pooled Prevalence of TD

The pooled prevalence of TD across the eight included studies is illustrated in Figure 2. In the forest plot, individual prevalence estimates are represented by squares, with sizes corresponding to the relative statistical weight assigned to each study. The prevalence estimates varied across the cohorts, reflecting differences in study populations and diagnostic approaches. The horizontal lines denote 95% CI and the diamond at the base represents the overall pooled prevalence derived from the random‐effects model.

**FIGURE 2:**
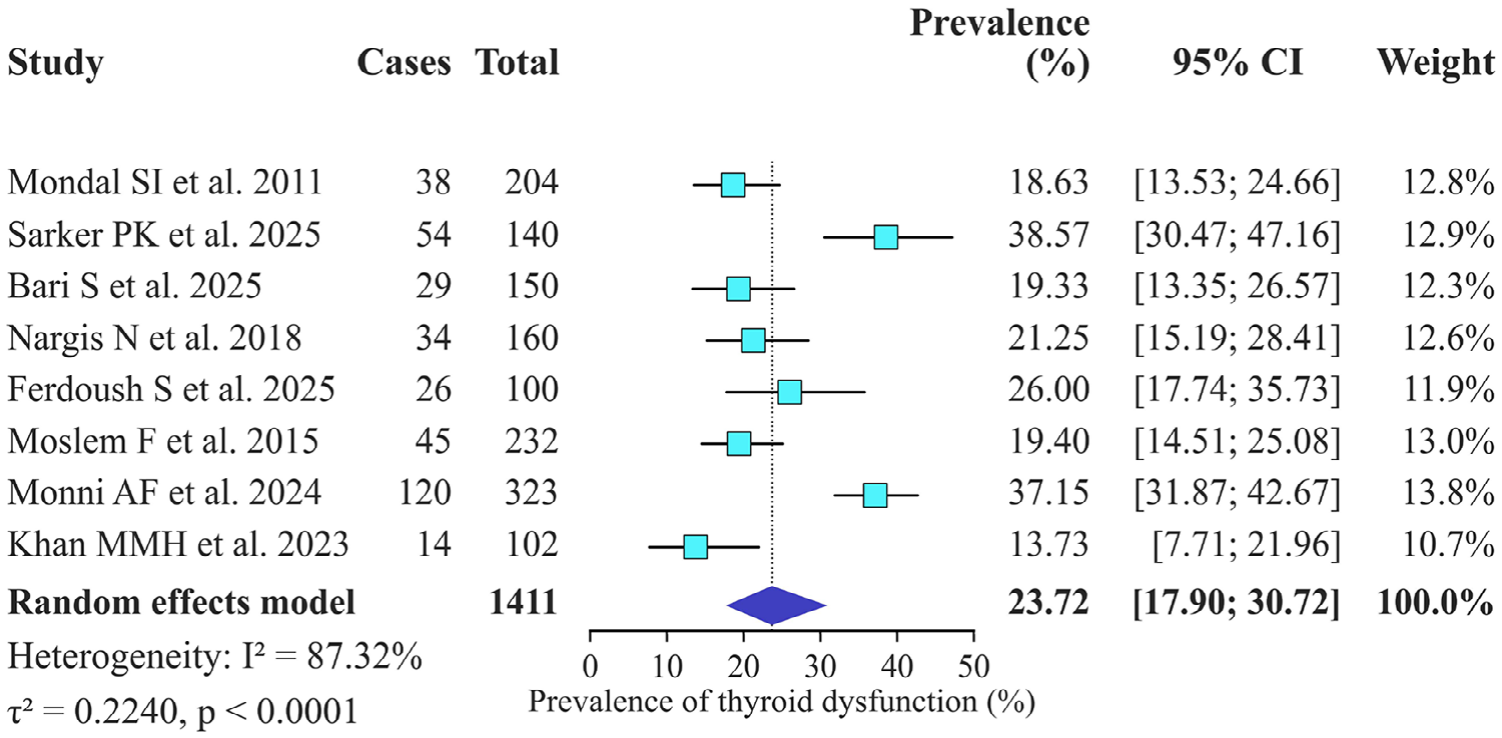
Forest plot representing the pooled prevalence of thyroid dysfunction among clinical populations in Bangladesh using a random‐effects model. CI, confidence interval.

The overall pooled prevalence of TD was 23.72% (95% CI: 17.90–30.72), indicating that nearly one‐quarter of the studied clinical population exhibited some form of thyroid abnormality. Substantial heterogeneity was observed (*I* ^2^ = 87.32%, *p* < 0.0001), suggesting significant between‐study variability likely associated with differences in participant demographics, underlying comorbidities, regional characteristics and laboratory assessment methodologies. Given this heterogeneity, the overall estimate should be interpreted as a pooled composite across clinically distinct populations (metabolic‐inflammatory and endocrine‐reproductive groups) rather than the prevalence within any single group; subgroup‐specific estimates (Section 3.5) offer more clinically interpretable figures for each population type.

### 3.5 Subgroup Analysis

Variations in the clinical profiles of the included populations contributed meaningfully to differences in prevalence (Figure S1). Studies focusing on endocrine‐reproductive populations reported a pooled prevalence of 19.14% (95% CI: 16.63–21.93) with no measurable between‐study heterogeneity (*I* ^2^ = 0%, *p* = 0.6834). Conversely, studies involving metabolic‐inflammatory disorder populations demonstrated a substantially higher pooled prevalence of 34.77% (95% CI: 28.52–41.59) with moderate heterogeneity (*I* ^2^ = 57.65%, *p* = 0.0943). The statistically significant subgroup difference (*p* < 0.0001) suggests that TD is more prevalent among individuals with metabolic or inflammatory conditions compared to endocrine‐reproductive populations. Diagnostic assessment strategies also influenced reported prevalence rates (Figure 3). Studies employing TSH combined with free hormone measurements (FT4 and/or FT3) showed a pooled prevalence of 28.31% (95% CI: 21.00–36.96; *I* ^2^ = 84.85%). In contrast, studies relying on TSH‐only or limited hormone assessments reported a lower prevalence of 18.31% (95% CI: 15.26–21.80; *I* ^2^ = 0%). The significant subgroup difference (*p* = 0.0140) suggests that broader biochemical testing may enhance the detection of TD cases that might otherwise remain unidentified.

**FIGURE 3:**
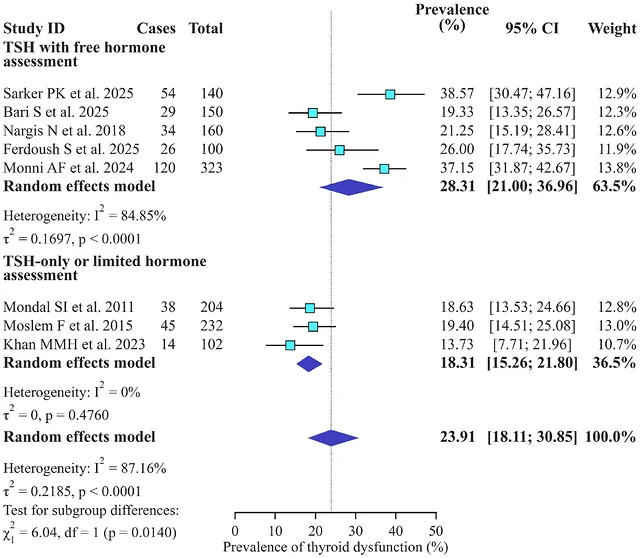
Forest plot comparing the prevalence of thyroid dysfunction based on diagnostic assessment methods (TSH with free hormones vs. TSH‐only or limited assessment). CI, confidence interval; TSH, thyroid‐stimulating hormone.

When stratified by the type of dysfunction, hypothyroidism emerged as the predominant abnormality (Figure 4). The pooled prevalence of hypothyroidism was 20.55% (95% CI: 15.22–27.14; *I* ^2^ = 86.92%), whereas the prevalence of hyperthyroidism was significantly lower at 4.93% (95% CI: 2.49–9.52; *I* ^2^ = 71.61%). This difference was statistically significant (*p* < 0.0001), confirming a clear predominance of hypothyroidism within the studied populations.

**FIGURE 4:**
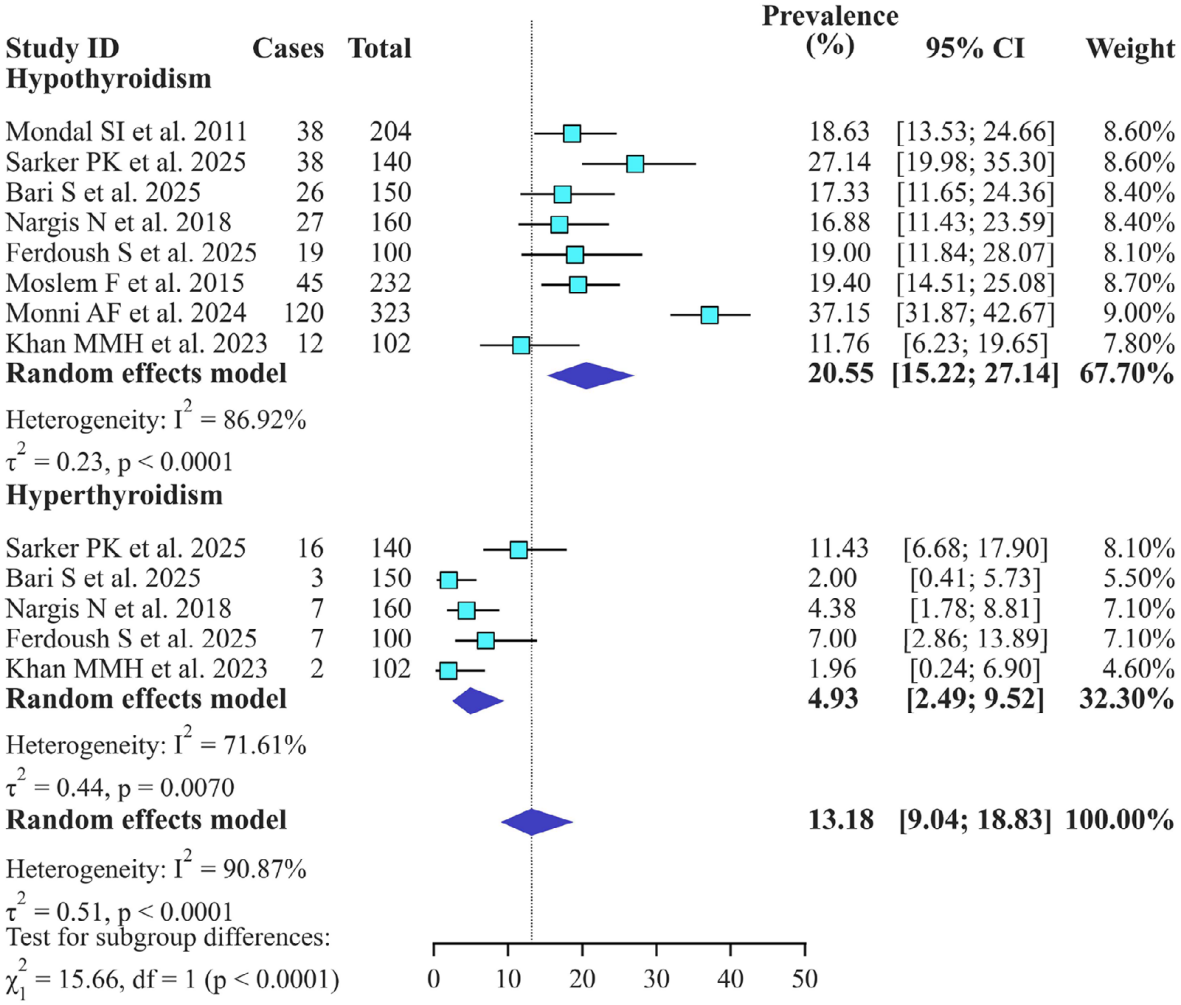
Forest plot showing the comparative pooled prevalence of hypothyroidism and hyperthyroidism across the included studies. CI, confidence interval.

Finally, stratification by sample size (using a median threshold of *n* = 155) revealed no meaningful differences in estimates (Figure 5). Studies with ≥155 participants showed a prevalence of 23.64% (95% CI: 15.55–34.23), nearly identical to the 23.66% (95% CI: 14.75–35.70) observed in studies with <155 participants. The absence of a significant subgroup difference (*p* = 0.9977) indicates that sample size had minimal influence on the pooled estimate, supporting the robustness of the findings.

**FIGURE 5:**
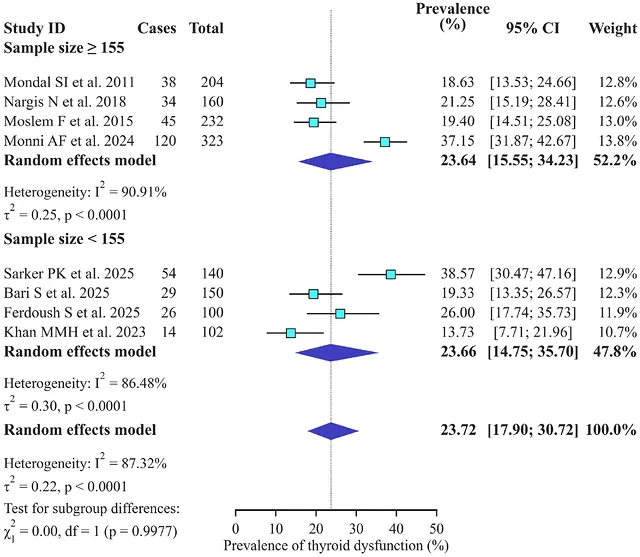
Forest plot evaluating the influence of study sample size (stratified by the median threshold of *n* = 155) on the pooled prevalence of thyroid dysfunction. CI, confidence interval.

### 3.6 Meta‐Regression Analysis

Univariable random‐effects meta‐regression analyses were performed to explore potential sources of between‐study heterogeneity in the pooled prevalence of TD (Table 2). Among the examined moderators, clinical population characteristics demonstrated the strongest association with prevalence variation. Studies involving metabolic‐inflammatory disorder populations reported significantly higher prevalence estimates compared to endocrine‐reproductive populations (*β* = 0.8461, *p* < 0.0001). Notably, this variable explained a substantial proportion of the observed between‐study heterogeneity (*R* ^2^ = 97.36%). Following adjustment for population type, residual heterogeneity was markedly reduced, suggesting that differences in underlying clinical conditions are a primary contributor to the variability across the included studies. Diagnostic assessment methods also showed a significant relationship with prevalence estimates. Studies employing comprehensive thyroid function assessments (TSH with FT4 and/or FT3) yielded higher prevalence estimates than those using limited evaluation approaches (*β* = 0.6168, *p* = 0.0349), accounting for 44.83% of the observed heterogeneity. Furthermore, a significant association was identified regarding the type of TD; hypothyroidism showed substantially higher prevalence estimates than hyperthyroidism (*β* = 1.5515, *p* < 0.0001), explaining 49.82% of the heterogeneity. In contrast, study sample size did not demonstrate a statistically significant association with prevalence estimates (*β* = 0.0024, *p* = 0.3387), with a minimal explanatory contribution (*R* ^2^ = 2.87%). Given the small number of included studies, this result should be interpreted with caution, as a single covariate can mechanically account for a large proportion of heterogeneity when *k* is limited. These findings suggest that clinical profiles, assessment methodologies, and disorder classification contribute more substantially to between‐study variability than methodological differences related to sample size.

**TABLE 2:** Univariable random‐effects meta‐regression analyses of factors associated with between‐study heterogeneity.

Variables	Coefficient (*β*)	SE	*p* value	*R* ^2^ (%)
Metabolic‐inflammatory disorder vs. endocrine‐reproductive populations	0.8461	0.138	<0.0001	97.36
Comprehensive thyroid function assessment vs. basic thyroid assessment	0.6168	0.2923	0.0349	44.83
Hypothyroidism vs. hyperthyroidism	1.5515	0.3586	<0.0001	49.82
Sample size	0.0024	0.0025	0.3387	2.87

### 3.7 Publication Bias and Sensitivity Analysis

The funnel plot (Figure 6) displayed a slight asymmetrical distribution of studies around the pooled prevalence estimate, with smaller studies showing greater variability. Although Peters’ regression test did not identify statistically significant asymmetry (*p* = 0.3844), the assessment of publication bias is limited by the small number of included studies (*n* = 8) and the substantial between‐study heterogeneity (*I* ^2^ = 87.2%). Therefore, the absence of detected publication bias should be interpreted cautiously.

**FIGURE 6:**
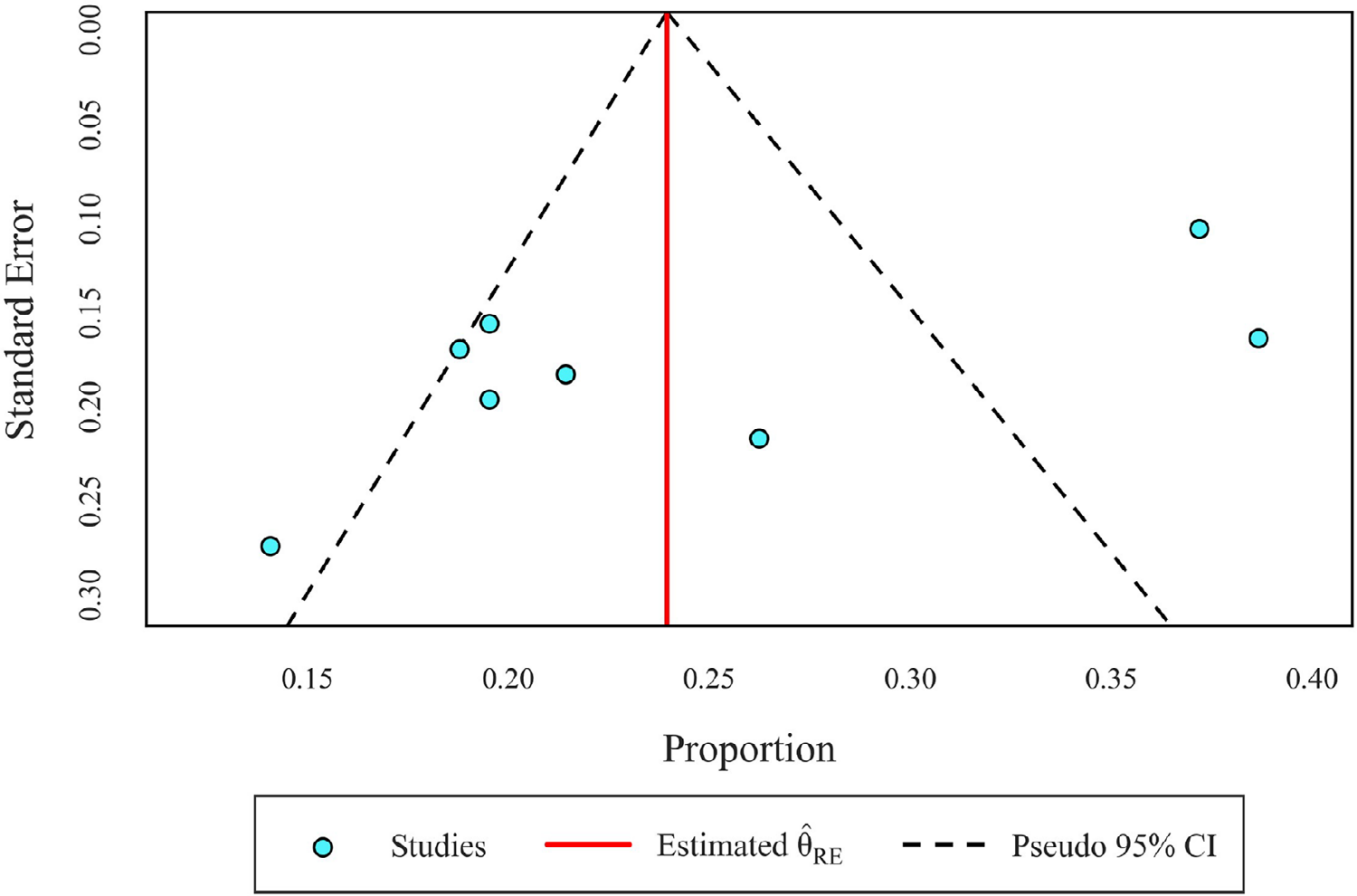
Funnel plot of the standard error by logit‐transformed prevalence. The asymmetrical distribution of studies was evaluated using Peters’ regression test to assess the potential for publication bias across the included investigations. CI, confidence interval.

The robustness of the pooled prevalence was further evaluated using a leave‐one‐out sensitivity analysis (Figure S2). The pooled prevalence estimates remained stable throughout the analysis, ranging from 22.19% (95% CI: 16.17–29.65) after the exclusion of [37] to 25.40% (95% CI: 18.51–33.80) after the exclusion of [38]. All recalculated estimates remained statistically significant (*p *< 0.001). This limited variation indicates that the overall results were not driven by any single investigation, supporting the consistency and robustness of the meta‐analysis findings despite the identified heterogeneity.

## 4 Discussion

The present systematic review and meta‐analysis establishes a high pooled prevalence of TD among clinical populations in Bangladesh at 23.72% (95% CI: 17.90–30.72). As this estimate pools clinically heterogeneous populations, it should be interpreted as an overall indicator of TD burden across Bangladeshi clinical settings rather than a prevalence applicable to any single patient group; the subgroup‐specific estimates offer more clinically actionable figures. This prevalence is substantially higher than the 10.03% reported in previous community‐based surveys in Dhaka, suggesting that clinical populations particularly those with metabolic and inflammatory disorders face a disproportionately higher burden of endocrine morbidity [44, 45, 46]. For instance, localized studies on women with polycystic ovary syndrome in Bangladesh have reported a TD prevalence of approximately 17% [47]. The elevated estimate in the current analysis likely reflects the inclusion of a broader spectrum of conditions, encompassing both overt and subclinical dysfunction, across diverse urban and rural clinical settings.

A primary finding of this study is the significant variation in prevalence across distinct clinical profiles. Patients with metabolic‐inflammatory disorders (e.g., diabetes mellitus, metabolic syndrome, and rheumatoid arthritis) demonstrated a markedly higher pooled prevalence of 34.77%. This finding aligns with the documented biological crosstalk between thyroid hormones and metabolic regulation [48, 49]. Insulin resistance, a hallmark of metabolic syndrome, has been mechanistically linked to elevated TSH levels and altered peripheral thyroid hormone deiodination [50]. Our results remain consistent with regional data from South Asia, which highlight that 20%–30% of diabetic patients harbor undiagnosed TDs [43, 51, 52, 53]. Such a high prevalence suggests that thyroid screening should be integrated as a routine component of metabolic disorder management in Bangladesh to mitigate the exacerbation of cardiovascular risks associated with these comorbid conditions [54]. In contrast, the endocrine‐reproductive subgroup exhibited a lower, yet more consistent, prevalence of 19.14%. While lower than the metabolic group, this figure represents a critical burden in the context of reproductive health. Thyroid hormones are essential for oocyte maturation and menstrual regulation; consequently, even subclinical dysfunction can significantly impair fertility and obstetric outcomes [55]. Stratification by the type of abnormality revealed a clear predominance of hypothyroidism (20.55%) over hyperthyroidism (4.93%). This 4:1 ratio is consistent with the global epidemiological transition from iodine‐deficiency‐related goiter to autoimmune‐driven thyroiditis, even in nations that have achieved iodine sufficiency [56]. Although Bangladesh has made significant strides in USI, the persistent high rates of hypothyroidism may reflect a rise in autoimmune thyroid disease (Hashimoto's thyroiditis) or environmental triggers associated with rapid urbanization [57, 58]. Similar patterns are observed in neighboring India and Pakistan, where hypothyroidism remains the primary thyroid abnormality in clinical settings [59, 60]. The meta‐regression analysis identified the diagnostic strategy as a significant moderator of reported prevalence. Studies employing comprehensive biochemical assessments (TSH combined with FT4 and/or FT3) reported a prevalence of 28.31%, whereas TSH‐only or limited testing yielded only 18.31%. This discrepancy suggests that a TSH‐centric screening approach may fail to detect nuanced cases, such as secondary hypothyroidism or T3‐toxicosis [61]. These results have direct clinical implications for the Bangladeshi healthcare system, suggesting that providers should adopt broader biochemical profiling particularly for high‐risk metabolic patients to ensure diagnostic accuracy and timely intervention. Despite the high between‐study heterogeneity (*I* ^2^ = 87.2%), our meta‐regression successfully explained over 97% of this variance through clinical population types. This indicates that the heterogeneity is not a result of methodological flaws but rather a reflection of the diverse clinical landscape in which TD manifests [62]. Addressing TD is fundamentally aligned with the SDG 3, which aim to ensure healthy lives and promote well‐being for all [63]. Early detection and management of thyroid disorders can prevent long‐term systemic complications, reduce the national healthcare economic burden, and contribute to broader health targets. Future research should prioritize longitudinal population‐based studies and the development of national thyroid screening guidelines to support evidence‐based, high‐impact clinical interventions in Bangladesh.

### 4.1 Limitations

Several limitations warrant consideration in the interpretation of this study. First, the geographical concentration of research in urban centers such as Dhaka and Sylhet may limit the generalizability of these findings to rural populations. Second, the lack of data regarding iodine status and thyroid autoantibodies (TPO‐Ab) in most studies precludes a definitive conclusion regarding the specific autoimmune or nutritional etiology of the observed dysfunction. Furthermore, the substantial between‐study heterogeneity identified is likely attributable to variations in clinical settings, diagnostic thresholds, and biochemical methodologies across the included cohorts. Additionally, the reliance on hospital‐based populations and the lack of standardized testing protocols in some studies may have influenced the pooled estimates. Finally, although no significant publication bias was detected via Peters’ regression test, the possibility of small‐study effects cannot be entirely excluded given the limited number of eligible studies. The small number of eligible studies (*k* = 8) limits the precision and generalizability of the pooled prevalence estimate and constrains the statistical power of meta‐regression analyses; findings from these analyses, including the high *R* ^2^ values, should therefore be interpreted as indicative rather than conclusive. Diagnostic criteria for TD varied across included studies, ranging from TSH‐only to TSH combined with total T4 or free hormones. This inconsistency in case definition may limit direct comparability of prevalence estimates across studies, despite our attempt to account for this variation through diagnostic‐strategy subgroup analysis.

## 5 Conclusions

This systematic review and meta‐analysis establishes TD as a major public health concern within the clinical landscape of Bangladesh, with a pooled composite estimate indicating substantial thyroid abnormality burden across diverse clinical settings most notably among patients with metabolic‐inflammatory conditions underscoring the need for population‐specific screening rather than a uniform prevalence assumption. Hypothyroidism was identified as the predominant disorder, occurring significantly more frequently than hyperthyroidism. A critical finding of this study is the “dual burden” of endocrine and systemic diseases, evidenced by a markedly higher prevalence of TD among patients with metabolic and inflammatory conditions. Although substantial variability was observed across studies likely influenced by differences in participant characteristics, underlying comorbidities, and diagnostic methodologies the consistent overall pattern underscores the clinical significance of these disorders. These findings suggest that current TSH‐centric screening may be insufficient and emphasize the urgent need for integrated metabolic screening and optimized biochemical evaluation, particularly within high‐risk groups. To mitigate the long‐term complications of untreated TD, it is recommended that healthcare providers integrate routine thyroid assessment into management protocols for patients with diabetes and metabolic syndrome. Furthermore, this study highlights the necessity for future nationwide, multicenter investigations using standardized protocols and autoimmune markers. Such research will be essential to providing representative national estimates and guiding evidence‐informed strategies for the screening, diagnosis, and management of thyroid disorders to optimize endocrine care in Bangladesh.

## Author Contributions

**Most. Rubaia Akter Rifat**: conceptualization, methodology, literature search, data extraction, quality assessment, writing – original draft, project administration. **Md. Raiyan Hasan Nabid**: formal analysis, software, data visualization, statistical synthesis, writing – review and editing. **Md. Tanjim**: literature search, data extraction, quality assessment, validation, writing – review and editing. **Nourin Sultana**: literature search, data extraction, quality assessment, resources, writing – review and editing. **Md. Moin Uddin Riyad**: conceptualization, methodology, validation, technical supervision, writing – review and editing. **Md. Kaderi Kibria**: conceptualization, methodology, supervision, data validation, critical revision of the manuscript and final approval.

## Funding

The authors have nothing to report.

## Ethics Statement

This study was a systematic review and meta‐analysis of previously published data; therefore, no ethical approval was required as no primary data were collected from human participants and no direct interaction with human or animal subjects occurred. All data used in this analysis were extracted from previously published, peer‐reviewed studies that had already obtained the necessary ethical approvals in their respective original studies.

## Conflicts of Interest

The authors declare no conflicts of interest.

## Supporting information




**Supporting Table 1:** NOS‐based quality evaluation of the eligible studies.
**Supporting Figure 1:** Forest plot illustrating the prevalence of thyroid dysfunction stratified by clinical profile such as endocrine‐reproductive populations versus metabolic‐inflammatory disorder populations.
**Supporting Figure 2:** Leave‐one‐out sensitivity analysis evaluating the influence of individual studies on the pooled prevalence of thyroid dysfunction among Bangladeshi clinical populations.

## Data Availability

The data underlying the results of this study are fully available within the manuscript.
