# Supplementary material for: Prevalence and Associated Factors of Thyroid Dysfunction Among Clinical Populations in Bangladesh: A Systematic Review and Meta‐Analysis
Source: Public Health Chall. 2026 Nov 7;5(4):e70446. doi: 10.1002/puh2.70446 (PMC13744620; doi:10.1002/puh2.70446)
Supplement: Supplementary file 1 [file PUH2-5-e70446-s001.docx]

**Table S1:** NOS - Based Quality Evaluation of the Eligible Studies

| **References** | **Representativeness** | **Response rate** | **Sample Size** | **Surveillance tool (2)** | **Comparability** | **Outcome Assessment (2)** | **Statistical test** | **Quality Score** |
| --- | --- | --- | --- | --- | --- | --- | --- | --- |
| Mondal SI, et al. 2011 | 1 | 0 | 1 | 2 | 1 | 2 | 1 | 8 |
| Sarker,P.K.,et al. 2025 | 1 | 0 | 1 | 2 | 0 | 2 | 1 | 7 |
| Bari S,et al. 2025 | 0 | 0 | 1 | 2 | 0 | 2 | 1 | 6 |
| Nargis N,et al. 2018 | 0 | 0 | 1 | 2 | 0 | 2 | 1 | 6 |
| Ferdoush S,et al. 2025 | 1 | 0 | 1 | 2 | 0 | 2 | 1 | 7 |
| Moslem F,et al. 2015 | 1 | 0 | 1 | 2 | 0 | 2 | 1 | 7 |
| Monni AF,et al. 2024 | 1 | 0 | 1 | 2 | 0 | 2 | 1 | 7 |
| Khan MMH,et al. 2023 | 1 | 0 | 1 | 2 | 0 | 2 | 1 | 7 |

**Figure S1:** Forest plot illustrating the prevalence of thyroid dysfunction stratified by clinical profile such as endocrine-reproductive populations versus metabolic-inflammatory disorder populations.

**Figure S2:** Leave-one-out sensitivity analysis evaluating the influence of individual studies on the pooled prevalence of thyroid dysfunction among Bangladeshi clinical populations.
